# Crystal structure of tris­(*N*-methyl­salicyl­aldiminato-κ^2^ *N*,*O*)chromium(III)

**DOI:** 10.1107/S2056989015023038

**Published:** 2015-12-06

**Authors:** Jessica Hilbert, Sven Kabus, Christian Näther, Wolfgang Bensch

**Affiliations:** a Institute of Inorganic Chemistry, Christian-Albrechts-University of Kiel, Max-Eyth-Strasse 2, 24118 Kiel, Germany

**Keywords:** crystal structure, chromium(III), *N*-methyl­salicylaldiminate, hydrogen bonding

## Abstract

The crystal structure of the title compound, [Cr(C_8_H_8_NO)_3_], is isotypic with the vanadium(III) analogue. The asymmetric unit consists of one Cr^3+^ cation and three *N*-methyl­salicylaldiminate anions. The metal cation is octa­hedrally coordinated by three *N*,*O*-chelating *N*-methyl­salicylaldiminate ligands, leading to discrete and neutral complexes. In the crystal, neighbouring complexes are linked *via* C—H⋯O hydrogen-bonding inter­actions into chains propagating parallel to the *c* axis.

## Related literature   

This structure determination was undertaken as part of a project intending to synthesise chromium-containing thio­stannates, for which no compounds are known to date. Instead, the title compound was isolated. Its structure is isotypic with the vanadium(III) analogue reported recently by us (Hilbert *et al.*, 2015 ▸). For the structures of similar discrete vanadium complexes with *N*-methyl­salicylaldiminate as ligand, see: Cornman *et al.* (1997 ▸).
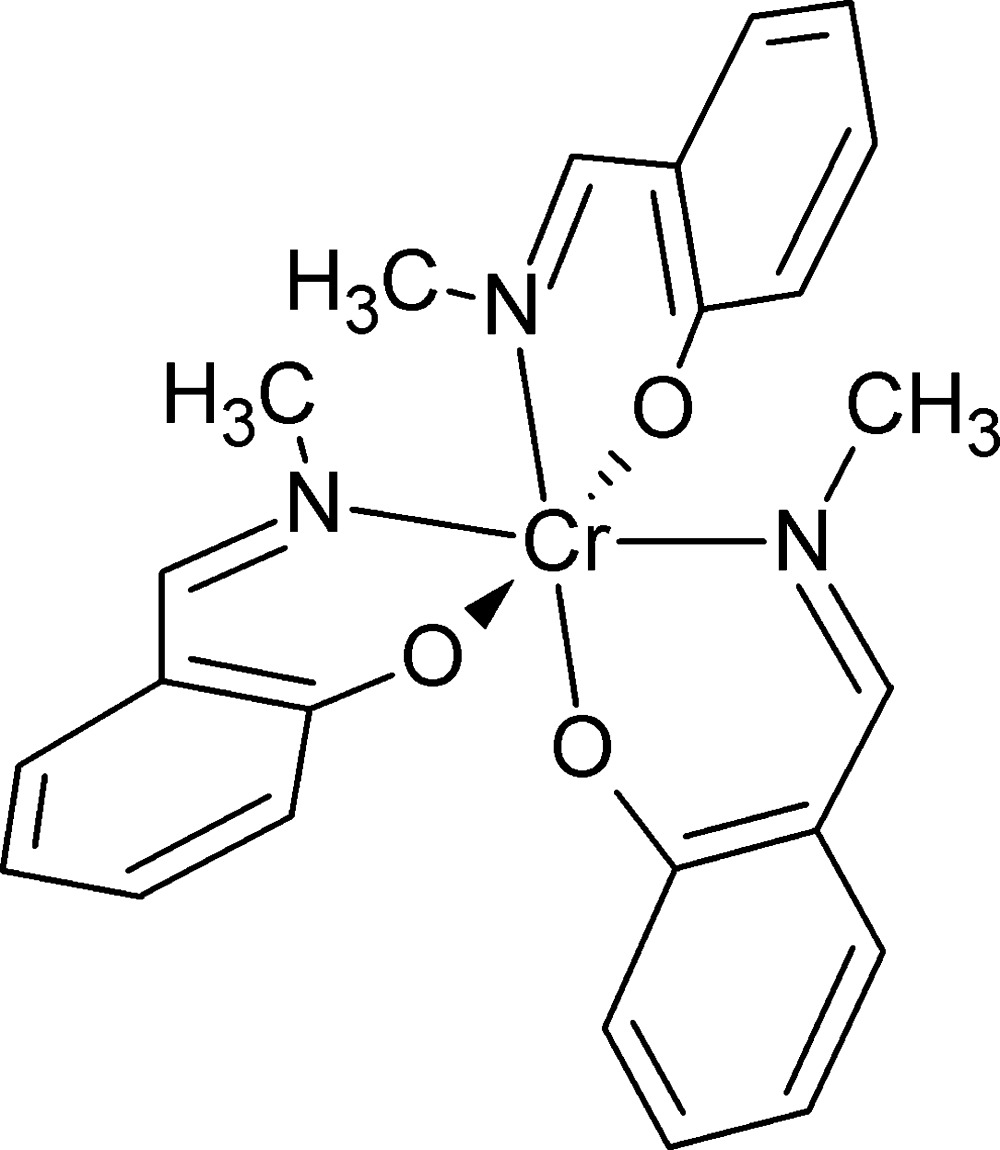


## Experimental   

### Crystal data  

[Cr(C_8_H_8_NO)_3_]
*M* *_r_* = 454.46
Monoclinic, 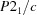

*a* = 7.7463 (2) Å
*b* = 25.4402 (8) Å
*c* = 11.1421 (3) Å
β = 102.659 (2)°
*V* = 2142.37 (11) Å^3^
*Z* = 4
Mo *K*α radiation
μ = 0.57 mm^−1^
*T* = 170 K
0.28 × 0.2 × 0.06 mm

### Data collection  

Stoe IPDS-1 diffractometer
Absorption correction: numerical (*X-SHAPE* and *X-RED32*; Stoe, 2008 ▸) *T*_min_ = 0.841, *T*_max_ = 0.972
25422 measured reflections
4665 independent reflections
3991 reflections with *I* > 2σ(*I*)
*R*_int_ = 0.030

### Refinement  

*R*[*F* ^2^ > 2σ(*F* ^2^)] = 0.033
*wR*(*F* ^2^) = 0.092
*S* = 1.07
4665 reflections
283 parameters
H-atom parameters constrained
Δρ_max_ = 0.32 e Å^−3^
Δρ_min_ = −0.41 e Å^−3^

### 

Data collection: *X-AREA* (Stoe, 2008 ▸); cell refinement: *X-AREA*; data reduction: *X-AREA*; program(s) used to solve structure: *SHELXS97* (Sheldrick, 2008 ▸); program(s) used to refine structure: *SHELXL2014* (Sheldrick, 2015 ▸); molecular graphics: *XP* in *SHELXTL* (Sheldrick, 2008 ▸) and *DIAMOND* (Brandenburg, 1999 ▸); software used to prepare material for publication: *publCIF* (Westrip, 2010 ▸).

## Supplementary Material

Crystal structure: contains datablock(s) I, global. DOI: 10.1107/S2056989015023038/wm5245sup1.cif

Structure factors: contains datablock(s) I. DOI: 10.1107/S2056989015023038/wm5245Isup2.hkl

**Fig. 1.**
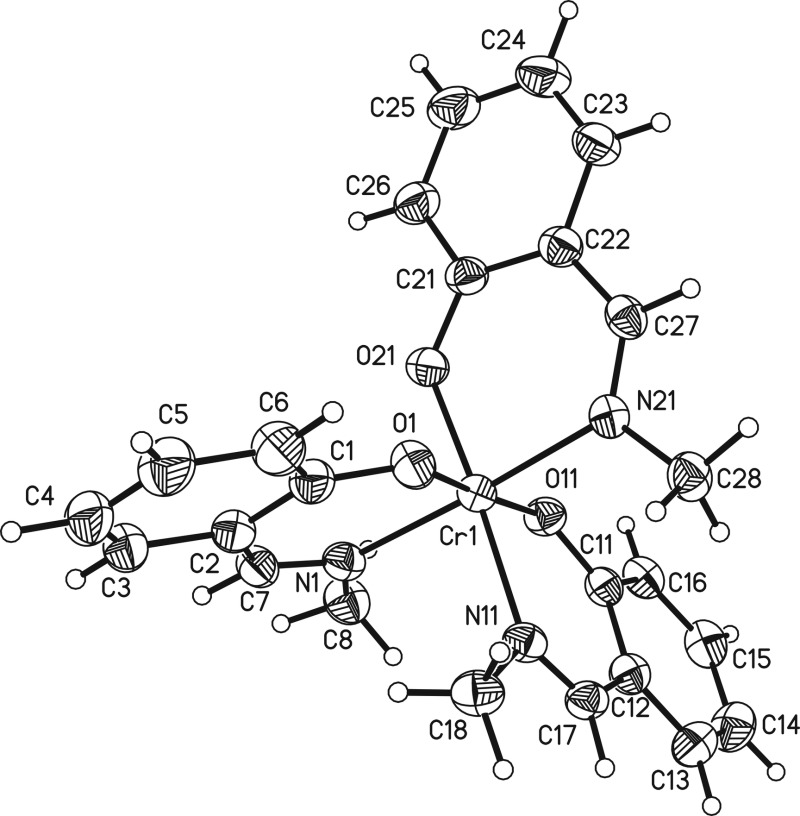
The molecular structure of the title compound. Displacement ellipsoids are drawn at the 50% probability level.

. DOI: 10.1107/S2056989015023038/wm5245fig1.tif
The mol­ecular structure of the title compound. Displacement ellipsoids are drawn at the 50% probability level.

**Fig. 2.**
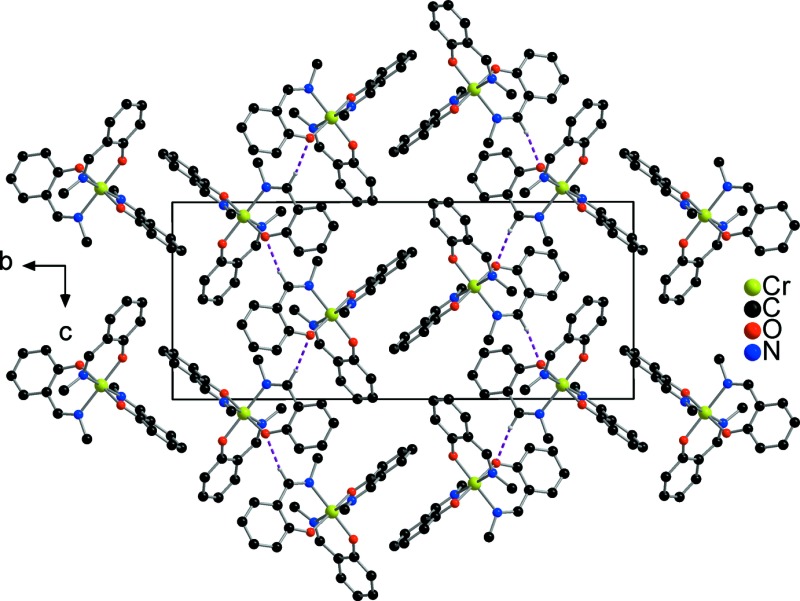
The crystal structure of the title compound in a view along [100]. C—H···O hydrogen bonds are shown as dashed lines. For clarity, all H atoms except those that participate in hydrogen bonding were omitted.

. DOI: 10.1107/S2056989015023038/wm5245fig2.tif
The crystal structure of the title compound in a view along [100]. C—H⋯O hydrogen bonds are shown as dashed lines. For clarity, all H atoms except those that participate in hydrogen bonding were omitted.

CCDC reference: 1439806

Additional supporting information:  crystallographic information; 3D view; checkCIF report

## Figures and Tables

**Table 1:** Hydrogen-bond geometry (Å, °)

*D*—H⋯*A*	*D*—H	H⋯*A*	*D*⋯*A*	*D*—H⋯*A*
C17—H17⋯O11^i^	0.95	2.44	3.3154 (19)	154

Symmetry code: (i) 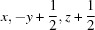.

## supplementary crystallographic information

### S1. Synthesis and crystallization

All chemicals were commercially available. The title compound was serendipitously obtained under solvothermal conditions. For the synthesis 66.6 mg (0.25 mmol) CrCl_3_·6H_2_O (Merck, 95%), 29.7 mg (0.25 mmol) Sn (Fluka, 99.9%), 24.1 mg (0.75 mmol) S (Alfa Aesar, 99.5%) and 134.2 mg (0.5 mol) *N*,*N*-ethyl­enebis(salicyl­imine) (Alfa Aesar, 99%) were mixed in a glass tube (inner volume 11 ml) with 1.5 ml methyl­amine (abcr, 40% aqueous solution) and 0.5 ml water. The reaction slurry was tempered at 398 K for one day. After cooling to room temperature the crystalline product (dark red blocks) was filtered off, washed with water and ethanol and dried over silica gel.

### S2. Refinement

The C-bound H atoms were positioned with idealized geometry (methyl H atoms were allowed to rotate but not to tip) and were refined with *U*_iso_(H) = 1.2*U*_eq_(C) (1.5 for methyl H atoms) using a riding model with C—H = 0.95 Å for aromatic H atoms and 0.98 Å for methyl H atoms.

### Crystal data

**Table:**

[Cr(C_8_H_8_NO)_3_]	*F*(000) = 948
*M**_r_* = 454.46	*D*_x_ = 1.409 Mg m^−^^3^
Monoclinic, *P*2_1_/*c*	Mo *K*α radiation, λ = 0.71073 Å
*a* = 7.7463 (2) Å	Cell parameters from 25422 reflections
*b* = 25.4402 (8) Å	θ = 1.6–27.0°
*c* = 11.1421 (3) Å	µ = 0.57 mm^−^^1^
β = 102.659 (2)°	*T* = 170 K
*V* = 2142.37 (11) Å^3^	Block, red
*Z* = 4	0.28 × 0.2 × 0.06 mm

### Data collection

**Table:**

Stoe IPDS-1 diffractometer	3991 reflections with *I* > 2σ(*I*)
Radiation source: fine-focus sealed tube	*R*_int_ = 0.030
phi–scans	θ_max_ = 27.0°, θ_min_ = 1.6°
Absorption correction: numerical (*X-SHAPE* and *X-RED32*; Stoe, 2008)	*h* = −9→9
*T*_min_ = 0.841, *T*_max_ = 0.972	*k* = −32→32
25422 measured reflections	*l* = −14→14
4665 independent reflections	

### Refinement

**Table:**

Refinement on *F*^2^	0 restraints
Least-squares matrix: full	Hydrogen site location: inferred from neighbouring sites
*R*[*F*^2^ > 2σ(*F*^2^)] = 0.033	H-atom parameters constrained
*wR*(*F*^2^) = 0.092	*w* = 1/[σ^2^(*F*_o_^2^) + (0.0557*P*)^2^ + 0.4287*P*] where *P* = (*F*_o_^2^ + 2*F*_c_^2^)/3
*S* = 1.07	(Δ/σ)_max_ < 0.001
4665 reflections	Δρ_max_ = 0.32 e Å^−^^3^
283 parameters	Δρ_min_ = −0.41 e Å^−^^3^

### Special details

**Table:**

Geometry. All esds (except the esd in the dihedral angle between two l.s. planes) are estimated using the full covariance matrix. The cell esds are taken into account individually in the estimation of esds in distances, angles and torsion angles; correlations between esds in cell parameters are only used when they are defined by crystal symmetry. An approximate (isotropic) treatment of cell esds is used for estimating esds involving l.s. planes.

### Fractional atomic coordinates and isotropic or equivalent isotropic displacement parameters (Å^2^)

**Table:**

	*x*	*y*	*z*	*U*_iso_*/*U*_eq_	
Cr1	0.61749 (3)	0.34539 (2)	0.42884 (2)	0.02599 (9)	
C1	0.7885 (2)	0.43007 (6)	0.59566 (15)	0.0326 (3)	
C2	0.6253 (2)	0.45158 (6)	0.61013 (15)	0.0329 (3)	
C3	0.6253 (3)	0.49641 (7)	0.68446 (16)	0.0392 (4)	
H3	0.5156	0.5110	0.6925	0.047*	
C4	0.7796 (3)	0.51944 (7)	0.74539 (18)	0.0455 (4)	
H4	0.7771	0.5493	0.7962	0.055*	
C5	0.9405 (3)	0.49834 (8)	0.73162 (19)	0.0468 (5)	
H5	1.0482	0.5142	0.7730	0.056*	
C6	0.9450 (3)	0.45480 (7)	0.65863 (18)	0.0415 (4)	
H6	1.0561	0.4412	0.6506	0.050*	
O1	0.80043 (15)	0.38808 (5)	0.52894 (11)	0.0343 (3)	
C7	0.4555 (2)	0.42962 (7)	0.55375 (15)	0.0334 (3)	
H7	0.3547	0.4475	0.5688	0.040*	
N1	0.42562 (18)	0.38859 (5)	0.48548 (12)	0.0302 (3)	
C8	0.2410 (2)	0.37274 (8)	0.43983 (18)	0.0387 (4)	
H8A	0.1627	0.3983	0.4667	0.058*	
H8B	0.2221	0.3379	0.4725	0.058*	
H8C	0.2147	0.3714	0.3498	0.058*	
C11	0.38538 (19)	0.25243 (6)	0.34655 (14)	0.0281 (3)	
C12	0.4286 (2)	0.22699 (6)	0.46229 (15)	0.0302 (3)	
C13	0.3594 (2)	0.17682 (7)	0.47646 (18)	0.0385 (4)	
H13	0.3867	0.1605	0.5550	0.046*	
C14	0.2529 (3)	0.15077 (7)	0.3791 (2)	0.0438 (4)	
H14	0.2106	0.1164	0.3894	0.053*	
C15	0.2085 (2)	0.17586 (7)	0.26552 (18)	0.0388 (4)	
H15	0.1346	0.1584	0.1979	0.047*	
C16	0.2704 (2)	0.22570 (7)	0.24959 (15)	0.0328 (3)	
H16	0.2349	0.2424	0.1718	0.039*	
O11	0.44612 (14)	0.29877 (4)	0.32539 (10)	0.0301 (2)	
C17	0.5348 (2)	0.25136 (6)	0.57088 (14)	0.0294 (3)	
H17	0.5476	0.2324	0.6458	0.035*	
N11	0.61359 (17)	0.29600 (5)	0.57646 (12)	0.0287 (3)	
C18	0.6981 (2)	0.31436 (7)	0.70060 (15)	0.0362 (4)	
H18A	0.6770	0.2888	0.7616	0.054*	
H18B	0.6480	0.3484	0.7160	0.054*	
H18C	0.8258	0.3181	0.7070	0.054*	
C21	0.6776 (2)	0.39336 (6)	0.19948 (15)	0.0305 (3)	
C22	0.8195 (2)	0.35940 (6)	0.19177 (15)	0.0317 (3)	
C23	0.9048 (3)	0.36486 (8)	0.09236 (17)	0.0408 (4)	
H23	1.0024	0.3428	0.0888	0.049*	
C24	0.8505 (3)	0.40106 (8)	0.00151 (18)	0.0493 (5)	
H24	0.9083	0.4038	−0.0651	0.059*	
C25	0.7087 (3)	0.43397 (8)	0.00799 (17)	0.0470 (5)	
H25	0.6700	0.4592	−0.0550	0.056*	
C26	0.6242 (3)	0.43040 (7)	0.10408 (16)	0.0384 (4)	
H26	0.5281	0.4533	0.1064	0.046*	
O21	0.59264 (15)	0.39170 (5)	0.28869 (11)	0.0342 (3)	
C27	0.8836 (2)	0.31882 (6)	0.28005 (15)	0.0302 (3)	
H27	0.9814	0.2989	0.2667	0.036*	
N21	0.82298 (17)	0.30641 (5)	0.37540 (12)	0.0283 (3)	
C28	0.9138 (2)	0.26427 (7)	0.45369 (15)	0.0351 (4)	
H28A	1.0136	0.2516	0.4207	0.053*	
H28B	0.8311	0.2353	0.4556	0.053*	
H28C	0.9577	0.2777	0.5373	0.053*	

### Atomic displacement parameters (Å^2^)

**Table:**

	*U*^11^	*U*^22^	*U*^33^	*U*^12^	*U*^13^	*U*^23^
Cr1	0.02573 (14)	0.02790 (14)	0.02495 (14)	−0.00027 (9)	0.00689 (10)	−0.00001 (9)
C1	0.0375 (9)	0.0301 (8)	0.0303 (8)	−0.0046 (7)	0.0078 (7)	−0.0021 (6)
C2	0.0394 (9)	0.0311 (8)	0.0289 (8)	0.0008 (7)	0.0093 (7)	0.0010 (6)
C3	0.0511 (11)	0.0333 (9)	0.0344 (9)	0.0033 (8)	0.0121 (8)	−0.0010 (7)
C4	0.0645 (13)	0.0341 (9)	0.0382 (10)	−0.0060 (9)	0.0122 (9)	−0.0060 (7)
C5	0.0529 (11)	0.0411 (10)	0.0443 (10)	−0.0143 (9)	0.0062 (9)	−0.0079 (8)
C6	0.0386 (9)	0.0411 (10)	0.0449 (10)	−0.0073 (8)	0.0096 (8)	−0.0057 (8)
O1	0.0300 (6)	0.0348 (6)	0.0388 (6)	−0.0032 (5)	0.0096 (5)	−0.0074 (5)
C7	0.0347 (8)	0.0357 (9)	0.0321 (8)	0.0048 (7)	0.0122 (7)	0.0004 (7)
N1	0.0282 (7)	0.0336 (7)	0.0301 (7)	0.0001 (5)	0.0090 (5)	0.0017 (5)
C8	0.0268 (8)	0.0446 (10)	0.0448 (10)	−0.0004 (7)	0.0086 (7)	−0.0032 (8)
C11	0.0244 (7)	0.0308 (8)	0.0296 (8)	0.0010 (6)	0.0072 (6)	−0.0019 (6)
C12	0.0252 (7)	0.0328 (8)	0.0327 (8)	0.0007 (6)	0.0064 (6)	0.0007 (6)
C13	0.0360 (9)	0.0347 (9)	0.0439 (10)	−0.0008 (7)	0.0067 (7)	0.0060 (7)
C14	0.0397 (10)	0.0343 (9)	0.0565 (12)	−0.0079 (7)	0.0083 (9)	−0.0014 (8)
C15	0.0304 (8)	0.0398 (9)	0.0454 (10)	−0.0040 (7)	0.0068 (7)	−0.0102 (8)
C16	0.0269 (7)	0.0397 (9)	0.0315 (8)	−0.0003 (6)	0.0056 (6)	−0.0051 (7)
O11	0.0322 (6)	0.0318 (6)	0.0257 (5)	−0.0021 (4)	0.0049 (4)	0.0007 (4)
C17	0.0271 (7)	0.0338 (8)	0.0275 (7)	0.0038 (6)	0.0064 (6)	0.0058 (6)
N11	0.0270 (6)	0.0348 (7)	0.0240 (6)	0.0011 (5)	0.0046 (5)	0.0017 (5)
C18	0.0379 (9)	0.0432 (9)	0.0253 (8)	−0.0029 (7)	0.0021 (7)	0.0011 (7)
C21	0.0331 (8)	0.0304 (8)	0.0288 (8)	−0.0057 (6)	0.0086 (6)	−0.0009 (6)
C22	0.0324 (8)	0.0331 (8)	0.0305 (8)	−0.0056 (6)	0.0089 (7)	−0.0035 (6)
C23	0.0446 (10)	0.0431 (10)	0.0393 (10)	−0.0030 (8)	0.0192 (8)	−0.0038 (8)
C24	0.0616 (13)	0.0546 (12)	0.0380 (10)	−0.0041 (10)	0.0247 (9)	0.0027 (9)
C25	0.0630 (13)	0.0457 (11)	0.0337 (9)	−0.0023 (9)	0.0136 (9)	0.0093 (8)
C26	0.0440 (10)	0.0356 (9)	0.0359 (9)	−0.0003 (7)	0.0098 (7)	0.0050 (7)
O21	0.0364 (6)	0.0345 (6)	0.0348 (6)	0.0048 (5)	0.0145 (5)	0.0061 (5)
C27	0.0254 (7)	0.0330 (8)	0.0328 (8)	−0.0017 (6)	0.0076 (6)	−0.0063 (6)
N21	0.0261 (6)	0.0286 (6)	0.0298 (7)	0.0003 (5)	0.0050 (5)	−0.0026 (5)
C28	0.0330 (8)	0.0376 (9)	0.0338 (8)	0.0077 (7)	0.0052 (7)	0.0013 (7)

### Geometric parameters (Å, º)

**Table:**

Cr1—O21	1.9318 (11)	C13—H13	0.9500
Cr1—O1	1.9337 (12)	C14—C15	1.391 (3)
Cr1—O11	1.9563 (11)	C14—H14	0.9500
Cr1—N1	2.0557 (13)	C15—C16	1.381 (3)
Cr1—N21	2.0705 (13)	C15—H15	0.9500
Cr1—N11	2.0752 (13)	C16—H16	0.9500
C1—O1	1.316 (2)	C17—N11	1.285 (2)
C1—C6	1.409 (2)	C17—H17	0.9500
C1—C2	1.419 (2)	N11—C18	1.471 (2)
C2—C3	1.410 (2)	C18—H18A	0.9800
C2—C7	1.440 (2)	C18—H18B	0.9800
C3—C4	1.370 (3)	C18—H18C	0.9800
C3—H3	0.9500	C21—O21	1.3077 (19)
C4—C5	1.396 (3)	C21—C26	1.413 (2)
C4—H4	0.9500	C21—C22	1.415 (2)
C5—C6	1.379 (3)	C22—C23	1.415 (2)
C5—H5	0.9500	C22—C27	1.437 (2)
C6—H6	0.9500	C23—C24	1.365 (3)
C7—N1	1.282 (2)	C23—H23	0.9500
C7—H7	0.9500	C24—C25	1.395 (3)
N1—C8	1.465 (2)	C24—H24	0.9500
C8—H8A	0.9800	C25—C26	1.375 (3)
C8—H8B	0.9800	C25—H25	0.9500
C8—H8C	0.9800	C26—H26	0.9500
C11—O11	1.3098 (19)	C27—N21	1.291 (2)
C11—C16	1.415 (2)	C27—H27	0.9500
C11—C12	1.416 (2)	N21—C28	1.462 (2)
C12—C13	1.407 (2)	C28—H28A	0.9800
C12—C17	1.445 (2)	C28—H28B	0.9800
C13—C14	1.380 (3)	C28—H28C	0.9800
			
O21—Cr1—O1	93.11 (5)	C12—C13—H13	119.2
O21—Cr1—O11	87.64 (5)	C13—C14—C15	118.72 (17)
O1—Cr1—O11	175.79 (5)	C13—C14—H14	120.6
O21—Cr1—N1	88.36 (5)	C15—C14—H14	120.6
O1—Cr1—N1	90.60 (5)	C16—C15—C14	120.98 (16)
O11—Cr1—N1	93.56 (5)	C16—C15—H15	119.5
O21—Cr1—N21	90.36 (5)	C14—C15—H15	119.5
O1—Cr1—N21	85.55 (5)	C15—C16—C11	121.43 (16)
O11—Cr1—N21	90.31 (5)	C15—C16—H16	119.3
N1—Cr1—N21	175.87 (5)	C11—C16—H16	119.3
O21—Cr1—N11	173.59 (5)	C11—O11—Cr1	131.59 (10)
O1—Cr1—N11	91.09 (5)	N11—C17—C12	126.65 (14)
O11—Cr1—N11	88.52 (5)	N11—C17—H17	116.7
N1—Cr1—N11	86.75 (5)	C12—C17—H17	116.7
N21—Cr1—N11	94.80 (5)	C17—N11—C18	115.80 (13)
O1—C1—C6	118.97 (16)	C17—N11—Cr1	125.89 (11)
O1—C1—C2	123.49 (15)	C18—N11—Cr1	118.19 (10)
C6—C1—C2	117.53 (15)	N11—C18—H18A	109.5
C3—C2—C1	119.56 (16)	N11—C18—H18B	109.5
C3—C2—C7	116.92 (16)	H18A—C18—H18B	109.5
C1—C2—C7	123.51 (15)	N11—C18—H18C	109.5
C4—C3—C2	121.68 (18)	H18A—C18—H18C	109.5
C4—C3—H3	119.2	H18B—C18—H18C	109.5
C2—C3—H3	119.2	O21—C21—C26	118.70 (15)
C3—C4—C5	118.94 (17)	O21—C21—C22	123.61 (15)
C3—C4—H4	120.5	C26—C21—C22	117.69 (15)
C5—C4—H4	120.5	C21—C22—C23	119.31 (16)
C6—C5—C4	120.81 (18)	C21—C22—C27	123.58 (14)
C6—C5—H5	119.6	C23—C22—C27	117.11 (16)
C4—C5—H5	119.6	C24—C23—C22	121.71 (18)
C5—C6—C1	121.47 (18)	C24—C23—H23	119.1
C5—C6—H6	119.3	C22—C23—H23	119.1
C1—C6—H6	119.3	C23—C24—C25	118.99 (17)
C1—O1—Cr1	130.42 (11)	C23—C24—H24	120.5
N1—C7—C2	127.12 (15)	C25—C24—H24	120.5
N1—C7—H7	116.4	C26—C25—C24	121.00 (18)
C2—C7—H7	116.4	C26—C25—H25	119.5
C7—N1—C8	117.74 (14)	C24—C25—H25	119.5
C7—N1—Cr1	124.64 (11)	C25—C26—C21	121.27 (17)
C8—N1—Cr1	117.55 (11)	C25—C26—H26	119.4
N1—C8—H8A	109.5	C21—C26—H26	119.4
N1—C8—H8B	109.5	C21—O21—Cr1	131.04 (11)
H8A—C8—H8B	109.5	N21—C27—C22	127.26 (15)
N1—C8—H8C	109.5	N21—C27—H27	116.4
H8A—C8—H8C	109.5	C22—C27—H27	116.4
H8B—C8—H8C	109.5	C27—N21—C28	117.13 (14)
O11—C11—C16	119.08 (14)	C27—N21—Cr1	124.09 (11)
O11—C11—C12	123.55 (14)	C28—N21—Cr1	118.66 (10)
C16—C11—C12	117.36 (15)	N21—C28—H28A	109.5
C13—C12—C11	119.80 (15)	N21—C28—H28B	109.5
C13—C12—C17	117.14 (15)	H28A—C28—H28B	109.5
C11—C12—C17	122.97 (15)	N21—C28—H28C	109.5
C14—C13—C12	121.63 (17)	H28A—C28—H28C	109.5
C14—C13—H13	119.2	H28B—C28—H28C	109.5

### Hydrogen-bond geometry (Å, º)

**Table:**

*D*—H···*A*	*D*—H	H···*A*	*D*···*A*	*D*—H···*A*
C17—H17···O11^i^	0.95	2.44	3.3154 (19)	154

Symmetry code: (i) *x*, −*y*+1/2, *z*+1/2.
