# Supplementary figures and images for: Crystal structure of tris­(N-methyl­salicyl­aldiminato-κ2 N,O)chromium(III)
Source: Acta Crystallogr E Crystallogr Commun. 2015 Dec 6;71(Pt 12):m247–8. doi: 10.1107/S2056989015023038 (PMC4719857; doi:10.1107/S2056989015023038)

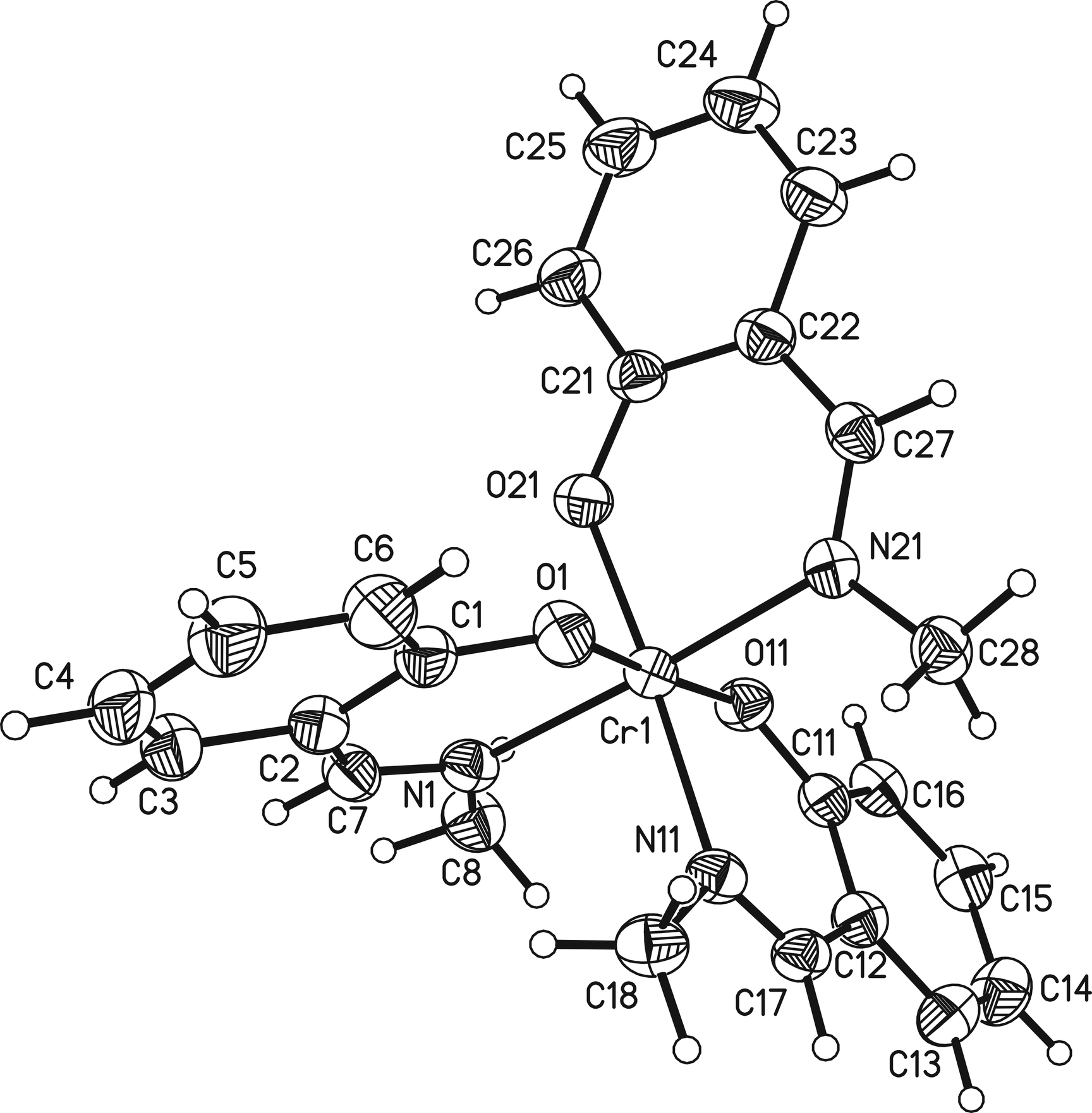

Supplement: Supplementary file 3 [file e-71-0m247-fig1.tif]

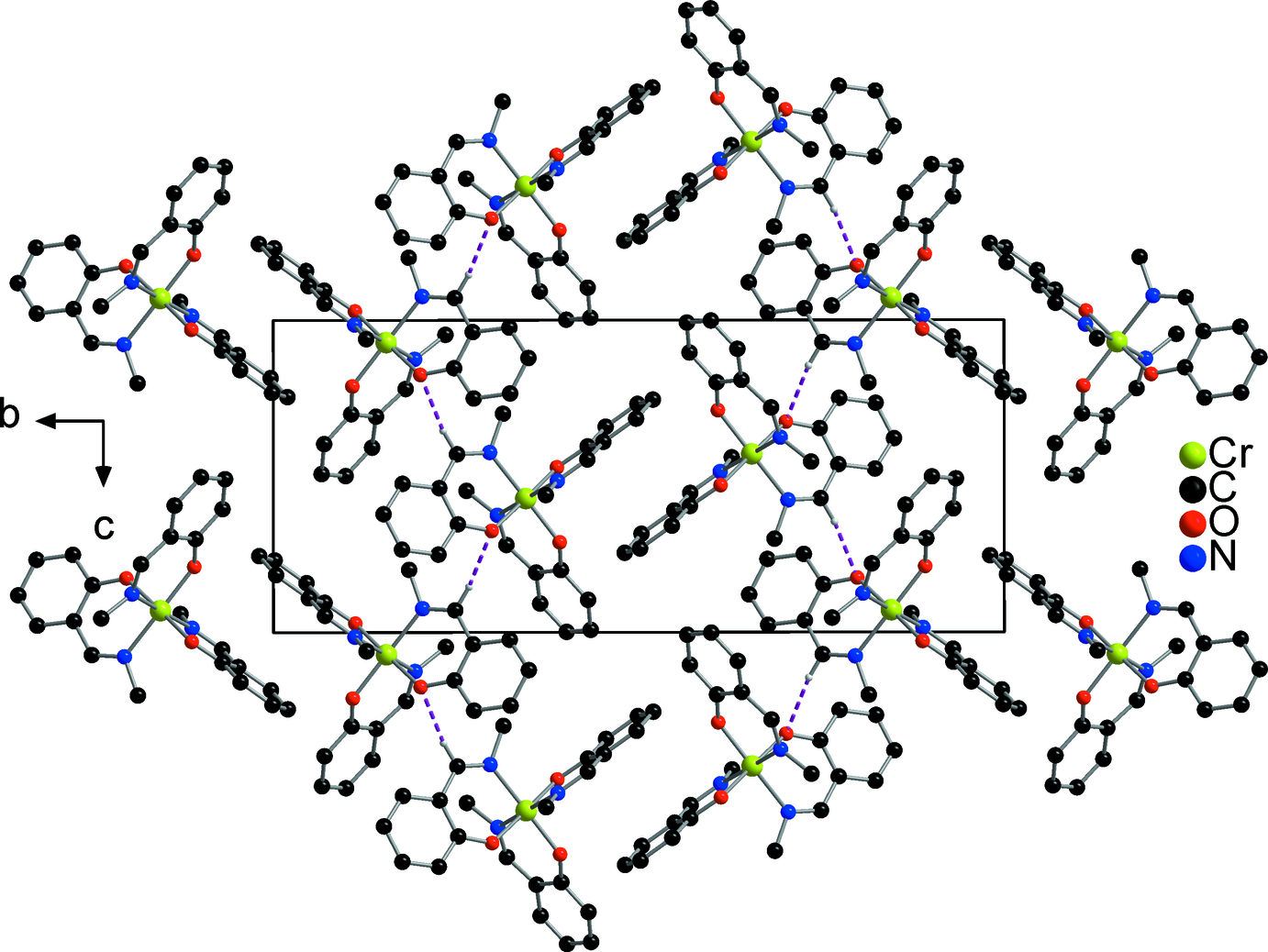

Supplement: Supplementary file 4 [file e-71-0m247-fig2.tif]
